# Multiscale Hierarchical Micro/Nanostructures Created by Femtosecond Laser Ablation in Liquids for Polarization-Dependent Broadband Antireflection

**DOI:** 10.3390/nano10081573

**Published:** 2020-08-11

**Authors:** Dongshi Zhang, Bikas Ranjan, Takuo Tanaka, Koji Sugioka

**Affiliations:** 1 Advanced Laser Processing Research Team, RIKEN Center for Advanced Photonics, Wako, Saitama 351-0198, Japan; dongshi17@126.com; 2 Innovative Photon Manipulation Research Team, RIKEN Center for Advanced Photonics, Wako, Saitama 351-0198, Japan; bikas.ranjan@riken.jp (B.R.); t-tanaka@riken.jp (T.T.); 3 Metamaterials Laboratory, RIKEN Cluster for Pioneering Research, Wako, Saitama 351-0198, Japan

**Keywords:** laser ablation in liquid, femtosecond laser, LIPSS, polarization dependent reflectance, hybrid LSFL/UHSFL nanostructure

## Abstract

In this work, we present the possibility of producing multiscale hierarchical micro/nanostructures by the femtosecond laser ablation of transition metals (i.e., Ta and W) in water and investigate their polarization-dependent reflectance. The hierarchical micro/nanostructures are composed of microscale-grooved, mountain-like and pit-rich structures decorated with hybrid laser-induced periodic surface structures (LIPSSs). The hybrid LIPSSs consist of low/high and ultrahigh spatial frequency LIPSSs (LSFLs/HSFLs and UHSFLs). LSFLs/HSFLs of 400–600 nm in a period are typically oriented perpendicular to the direction of the laser polarization, while UHSFLs (widths: 10–20 nm and periods: 30–50 nm) are oriented perpendicular to the curvatures of LSFLs/HSFLs. On the microstructures with height gradients, the orientations of LSFLs/HSFLs are misaligned by 18°. On the ablated W metasurface, two kinds of UHSFLs are observed. UHSFLs become parallel nanowires in the deep troughs of LSFLs/HSFLs but result in being very chaotic in shallow LSFLs, turning into polygonal nanonetworks. In contrast, chaotic USFLs are not found on the ablated Ta metasurfaces. With the help of Fourier transform infrared spectroscopy, it is found that microgrooves show an obvious polarization-dependent reflectance at wavelengths of 15 and 17.5 μm associated with the direction of the groove, and the integration of microstructures with LSFs/HSFLs/UHSFLs is thus beneficial for enhancing the light absorbance and light trapping in the near-to-mid-infrared (NIR-MIR) range.

## 1. Introduction

Femtosecond laser ablation (fs-LA) is a versatile technique that enables the production of a large variety of surface structures [1,2,3,4,5], and the structures’ diversity can be further enriched in combination with other techniques [6,7]. Laser-induced periodic surface structures (LIPSSs) [1,8,9,10,11,12,13] are the most typical nanoscale structures that are uniquely achievable by fs-LA, whose periods can be manipulated from tens of nm to hundreds of nm by changing the laser properties [14], processing parameters [8] and ablation environments [15]. Generally, LIPSSs are categorized into low and high spatial frequency LIPSSs (LSFL/HSFL) according to the ratio of LIPSS periods (Λ) to the fs laser wavelength (λ) [8]. The periods of LSFLs and HSFLs are defined as ranging from about the wavelength to half of the laser wavelength (λ/2 ≤ Λ_LSFL_ ≤ λ) and less than half of the wavelength (Λ_HSFL_ < λ/2), respectively [8,16]. Recently, our group has shown the necessity to define sub-100 nm [17,18,19,20] periods as a new category of ultrahigh spatial frequency LIPSSs (UHSFLs) because (1) UHSFLs whose periods are as small as 40 nm [17,21] are very difficult to form on semiconductors (so far, only two reports on Si) when compared with normal HSFLs with periods in the range of 100–200 nm [15,22,23]; (2) The periods of UHSFLs prepared on metals are much smaller than normal HSFLs with periods of hundreds of nm [24,25,26]. For example, Bosen et al. prepared homogeneous UHSFLs (periods: 70–90 nm) on Ti by fs-LA in air (λ = 790 nm, τ = 30–160 fs and υ = 1 kHz) [27,28,29] and found that such fine structures can be easily destroyed during the friction tests in two different lubricating oils [30]. Sedao et al. revealed the role of surface melting and resolidification in the formation of UHSFLs of 70–90 nm in a period on Ni [31].

Hierarchical LSFLs/UHSFLs structures are another kind of typical and unique structure only obtainable on transition metals (unavailable on semiconductors) via fs-LA. Abou–Saleh et al. found that UHSFLs were located between LSFLs on the Cr surface treated by fs-LA and proposed that spallation-induced roughness was a key factor in triggering the formation of UHSFLs during multi-pulse fs-LA [32]. Based on comprehensive experiments of fs-LA in acetone, our group demonstrated the material-dependent formation of hierarchical UHSFL/LSFL nanostructures on the group IVB−VIB transition metals (e.g., Ti, V, Nb, Ta, Mo and W) [18]. On group VIII (e.g., Fe, Pd, Pt and Ni) and IB/IB−IIB (e.g., Au, Ag, Cu and CuZn) transition metals or alloy, only LSFLs and hole-rich microstructures, rather than the hierarchical LSFLs/UHSFLs, were yielded [18]. The orientations of UHSFLs are commonly considered to be parallel to the direction of laser polarization [27]. In 2011, Vincenc Obona et al. reported that HSFLs were not dependent on the polarization vector on stainless steel [33]. Our group recently confirmed that Marangoni bursting was the predominant factor to determine the orientations of UHSFLs during fs-LA in liquids (fs-LAL) and clarified that UHSFLs were perpendicular to the curvatures of LSFLs [18]. 

Considering the uniqueness of LSFLs/UHSFLs obtained by fs-LA, it is highly desirable to integrate them onto microstructures in order to construct multiscale hierarchical micro/nanostructures, which will promise the generation of specific functions. To date, although many hierarchical micro/nanostructures [34,35] have been created and some of them have been applied in cellular behavior control [36,37], color imprinting [38], antireflectance [35,39,40] and superhydrophobicity/superoleophobicity [41,42,43], a report on the preparation and application of LSFLs/UHSFLs-containing hierarchical micro/nanostructures is still lacking.

In this work, aimed to fill this gap, we demonstrate the possibility of developing multiscale hierarchical structures via fs-LA of W and Ta in water and investigate how such hierarchical microstructures influence the optical properties. The surface morphologies are analyzed by scanning electron microscopy (SEM), energy dispersive spectrometer (EDS) and three-dimensional (3D) confocal microscopy. Two different hierarchical micro/nanostructures on ablated Ta and W samples are chosen as representatives to demonstrate the impact of hierarchical microstructures on polarization-dependent reflectance. 

## 2. Materials and Methods

An fs laser system (FCPA μ Jewel D-1000-UG3, IMRA America Inc., Ann Arbor, MI, USA) was used for laser ablation. The pulse duration, wavelength and repetition rate were 457 fs, 1045 nm and 100 kHz, respectively. The laser power was set at 600 mW. The linearly polarized laser beam was focused by a 20× objective lens (numerical aperture, 0.42; Mitutoyo, Kawasaki, Japan). The laser spot size was 3.4 μm, so the fluence was calculated to be 66.12 J/cm^2^. Ta (10 × 10 × 1 mm^3^) and W (10 × 10 × 2 mm^3^) targets were placed inside a glass container (Φ = 45 mm; 20 mm height) filled with 8 and 10 mL of water. The liquid thickness between the air-water interface and the ablated surface was kept at 5 mm. During fs-LAL, a syringe was used to remove the persistent bubbles adhering to the substrates from time to time. Otherwise, the persistent bubbles would cause a severe reflection and refraction of laser pulses, resulting in an inefficient ablation [44]. A line-by-line scanning method was employed with a line interval of 5 μm. The scanning direction was perpendicular to the light polarization direction. SEM (Quattro ESEM, Thermo Fisher Scientific, Tokyo, Japan) equipped with an EDS module and a confocal laser scanning microscope was used to characterize the structures formed on the ablated substrates. Fourier transform infrared (FTIR) spectroscopy (FT/IR-6300, JASCO, Tokyo, Japan) was used to test the polarization-dependent reflectance. 

## 3. Results and Discussion

### 3.1. SEM Characterization of Hierarchical Micro/Nanostructure Morphologies

**Figure 1:**
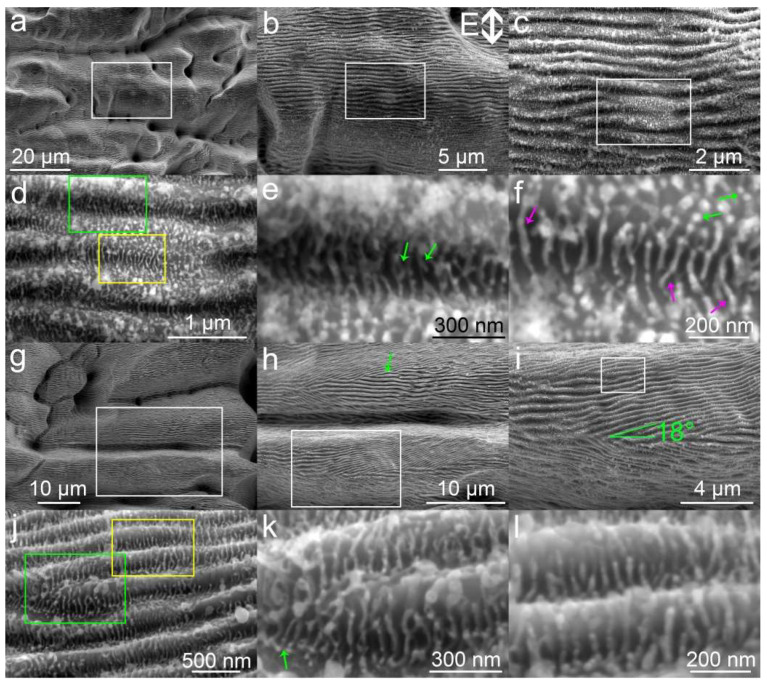
Scanning electron microscopy (SEM) images of hierarchical micro/nanostructures obtained by fs-LA of Ta in water. (**a**,**g**) Two typical low magnification images showing different kinds of microstructures at different regions. Light polarization direction is shown in (**b**). (**b**–**d**,**h**–**j**) are images magnified from the white rectangles of (**a**–**c**,**g**–**i**), respectively. (**e**/**f**,**k**/**l**) are images magnified from the green and yellow rectangles in (**d**,**j**), respectively.

Figure 1a displays the typical microstructures obtained by fs-LA of Ta in water. The microstructures are not uniform, consisting of microholes, curved troughs and mountain-like bump structures. The formation of curved troughs is attributed to the refraction and reflection by the bubbles generated during fs-LAL [17]. The diameter of microholes ranges from 2 to 5 μm. Enlarged images (Figure 1b,c) indicate that LIPSS nanostructures with periods of 400–600 nm are located on all microstructures. Although LIPSSs observed on the microstructures are categorized into the mixture of LSFLs/HSFLs (according to the definition of LSFL (λ/2 ≤ Λ_LSFL_ ≤ λ) and HSFL (Λ_HSFL_ < λ/2), together with the laser wavelength of 1045 nm used for fs-LAL), the formation mechanism should be the same due to the small difference in the period. The orientations of LSFLs/HSFLs are perpendicular to the direction of light polarization. Bifurcations of LSFLs can be obviously seen on the microstructures (Figure 1c). The depths of bifurcated LSFLs/HSFLs are much shallower than other parts (Figure 1d). A large amount of UHSFLs of 30–50 nm per period are located in the troughs and joint regions of LSFLs/HSFLs. The periods of UHSFLs are much narrower than those prepared on Ti by fs-LA in air [27,28,29]. In a relatively deeper trough of LSFLs/HSFLs, UHSFLs are characterized by extra stretched nanowires from their middle parts (pointed out by green arrows in Figure 1e), so that it is difficult to differentiate them from main UHSFLs (Figure 1e). In contrast, UHSFLs at the shallow region are much easier to identify, and some UHSFLs are decorated with nanodots (pointed out by green arrows in Figure 1f). Left-bent, right-bent and zigzag UHSFLs (pointed out by pink arrows) are all observed (Figure 1f), indicating that an extra turbulent factor is triggered during the formation of UHSFLs. 

Figure 1g demonstrates the possibility of producing microgrooves decorated with both tilted and horizontal LSFLs/HSFLs (magnified images are also shown in Figure 1h,i). The orientation of LSFLs/HSFLs pointed out by a green arrow is perpendicular to the direction of light polarization (Figure 1h), while the tilted LSFLs/HSFLs deviate by 18° from the microgroove direction, as expected (Figure 1i), resembling the herringbone structures on copper surfaces obtained by s-polarized fs laser ablation at a large incident angle [45]. Schwarz et al. reported that an inclined fs laser can significantly alter the orientations of LSFLs on fused silica [46]. Zheng et al. also proposed that the inclined angle at the boundary of a Gaussian beam can lead to the formation of slantwise-oriented LIPSSs [47]. Hence, the formation of herringbone structures shown in Figure 1i is deemed to be caused by an inclined fs laser ablation arising from bubble reflection/refraction [17]. A higher magnification of LSFLs/HSFLs structures allows for the observation of the states of UHSFLs on the side walls of LSFLs/HSFLs, as shown in Figure 1j–l. The orientations of UHSFLs depend on the LSFLs/HSFLs where they are located. At the tip of LSFLs/HSFLs where LSFLs/HSFLs change their curvature, UHSFLs can be even aligned from left to right (indicated by a green arrow in Figure 1k). On the ridge of LSFLs/HSFLs, UHSFLs are always aligned perpendicular to the ridge of LSFLs (Figure 1l) along the ridge’s inclination. This finding is in accordance with our previous report, which showed that USHFLs are not parallel to the direction of light polarization but strongly related to the LSFLs/HSFLs curvatures [18]. The simultaneous formation of LSFLs/HSFLs may originate from the excitation and interference of counter-propagating surface plasmons with extreme wavenumbers during fs-LAL [48], while the formation of UHSFLs is attributed to Marangoni bursting during the formation of LSFLs/HSFLs molten layers [18].

**Figure 2:**
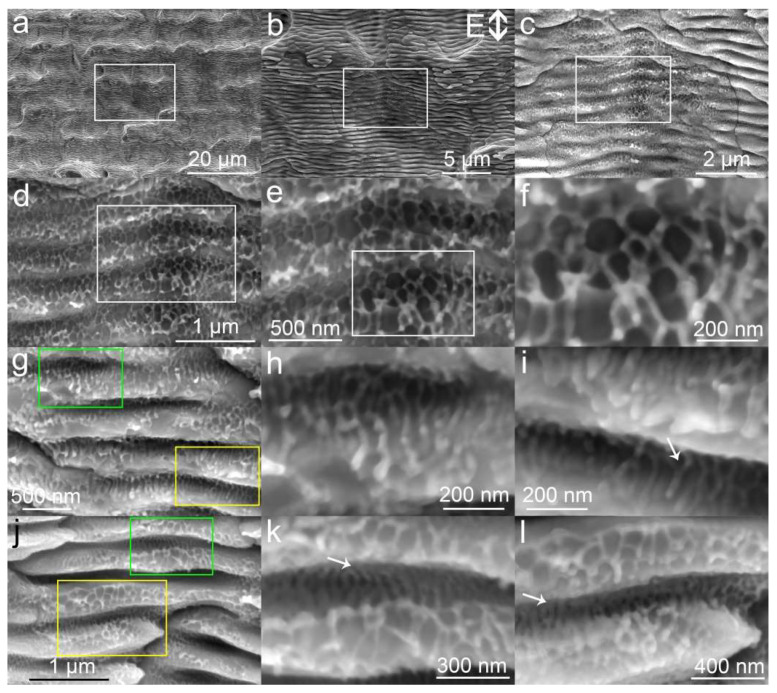
(**a**) SEM images of hierarchical micro/nanostructures obtained by fs-LA of W in water. The light polarization direction is shown in (**b**). (**b**–**f**) are magnified images from the white rectangle regions in (**a**–**e**), respectively. Parallel UHSFLs are indicated by white arrows in (**i**,**k**,**l**). (**g**,**j**) Two typical hierarchical LIPSS nanostructures with different states of UHSFLs. (**h**/**i**,**k**/**l**) are magnified images from the green and yellow rectangle regions in (**g**,**j**), respectively.

The structures obtained by fs-LAL of W in water show different morphologies from Ta. Many shallow microcraters (Figure 2a) are created on the W surface. All microstructures are decorated with LSFLs/HSFLs of 400–600 nm per period. The orientations of LSFLs/HSFLs are perpendicular to the direction of light polarization (Figure 2b). The LSFLs/HSFLs which are located at the bottom of craters are much shallower than those on the side part of craters, as shown in Figure 2c. At the bottom of the craters with shallow LSFLs/HSFLs (Figure 2d), UHSFLs become very chaotic, interconnecting with each other to form nanowire networks with various polygonal patterns (Figure 2e). The joint points of the polygonal UHSFLs networks are featured by ejected particles (Figure 2f). On the side ridge of the crater where deeper LSFLs/HSFLs are created (Figure 2g), UHSFLs are not so chaotic. UHSFLs are only interconnected in the deepest region of LSFL but become parallel at the outer space of LSFLs/HSFLs (Figure 2h magnified from the green rectangle in Figure 2g). In the yellow region of Figure 2g, UHSFLs with periods of 30–50 nm are parallel to each other, and their orientations are not parallel to the direction of light polarization but perpendicular to the curvatures of the underlying LSFLs/HSFLs (Figure 2i). In the regions where LSFLs/HSFLs have both deep troughs and inclined ridges (Figure 2j), UHSFLs are ordered in different ways. UHSFLs are highly ordered in the deep region of LSFLs/HSFLs, while they become very disordered on the outermost inclined ridges (indicated by white arrows in Figure 2k,l). This means that some factors that can arouse the disturbance of UHSFLs are triggered during the solidification of UHSFLs but that such turbulent factors have a limited influence on the deep troughs of LSFLs/HSFLs. The turbulent effect generated during fs-LAL of W is much stronger than that generated during fs-LAL of Ta. Similarly, Zhao et al. observed the formation of chaotic nanospikes (diameter 10–100 nm, length up to 250 nm) between LSFLs after the single-pulse fs laser irradiation of W in air [49]. Although the mechanism is still unclear and deserves further investigations, we speculate the following: W is a material with a high imaginary part of permittivity (ε″: 32.58 at 1060 nm [50]) that enables a very high absorbance of infrared light. Considering that an infrared pulse laser (wavelength: 1045 nm) was used for fs-LAL, the as-prepared surface structures effectively absorbed the beam energy, which caused an increase in the liquid temperature near the structures and may also have aroused additional magnetic and electrical effects near the surface structures [50]. In consequence, the orientations of UHSFLs were greatly disturbed during their solidification.

To compare the difference between our results and previous reports, LIPSSs obtained on Ta and W by fs-LA in both air and liquids are summarized in Table 1. In our previous report, we showed that, under the same ablation condition, the periods of LIPSSs (including both LSFLs/HSFLs and UHSFLs) obtained by fs-LAL in acetone [18] were almost the same as those produced by fs-LA in water shown in this work. Studies on Ta-LIPSS are very limited. Barmina et al. obtained LSFL/UHSL by fs laser ablation in water, but the SEM images of structures shown in their work were captured from an inclined angle, making it very hard to identify the periods of LSFLs [51]. Jorge–Mora et al. produced LSFLs on Ta surfaces by fs-LA in air with LIPSS periods which were slightly smaller than the laser wavelength (λ = 1030 nm vs. Λ = 780 nm) [52] but much larger than the periods (370–600 nm) of Ta-LIPSSs obtained in liquids (this work and ref. [18]). This means that liquid is beneficial for achieving narrower periods of LIPSSs, a trend that is also confirmed by the summary of the periods of Si-LIPSSs obtained in different environments [15]. Recently, Kudryashov et al. proposed that the factors of the squared optical refraction index (*n*) of environments can be used to estimate the environment-dependent periods (Λ) of LIPSSs following the trend of Λ ∝ 1/*n*^2^, which indicated that liquids with a higher refraction index than air normally produced smaller LIPSS periods [48]. The refractive indices of air, water, ethanol and acetone are 1, 1.33, 1.36 and 1.36, respectively, which would give ratios of LIPSS periods in air/water/ethanol/acetone (Λ_air_/Λ_water_/Λ_ethanol_/Λ_acetone_) of 1/1.33^2^/1.36^2^/1.36^2^. According to the fact that 780 nm is the period of Ta-LIPSS obtained by fs-LA in air at λ = 1030 nm, the periods of Ta-LIPSSs obtained in water and ethanol/acetone at the wavelength of 1030 nm should be 441 and 421 nm, respectively. This calculation fits well with the period range of 400–600 nm experimentally obtained by fs-LA in water and acetone at a close wavelength of 1045 nm, as presented in this work and our previous work [18]. Hence, it can be concluded that the theoretical trend of Λ ∝ 1/*n*^2^ proposed by Kudryashov et al. [48] is at least applicable to some cases. 

Compared to Ta-LIPSSs, W-LIPSSs have been studied much more. Roughly, the LIPSS periods obtained by fs-LAL in liquids such as ethanol and water are smaller than those obtained in air (Table 1). However, varying laser parameters such as pulse duration, wavelength, repetition rates and laser fluences can significantly change the periods of W-LIPSSs in an air environment. Some of W-LIPSSs’ periods are within the period range of W-LIPSSs obtained in liquids. The most noteworthy involves using an fs laser with an ultrahigh repetition rate of up to 80 MHz: the periods of W-LIPSSs are as small as 150–185 nm [53], equal to half of the W-HSFL periods obtained in liquids [18,54,55,56]. Similarly, LAL of Si in oil at a repetition rate of 90 MHz allows for the formation of abnormal LIPSSs with sub-100 nm periods [21]. The reason for this phenomenon is unknown, and it deserves a much deeper investigation. However, it is not within the scope of the present work.

### 3.2. 3D Morphology Characterization

**Figure 3:**
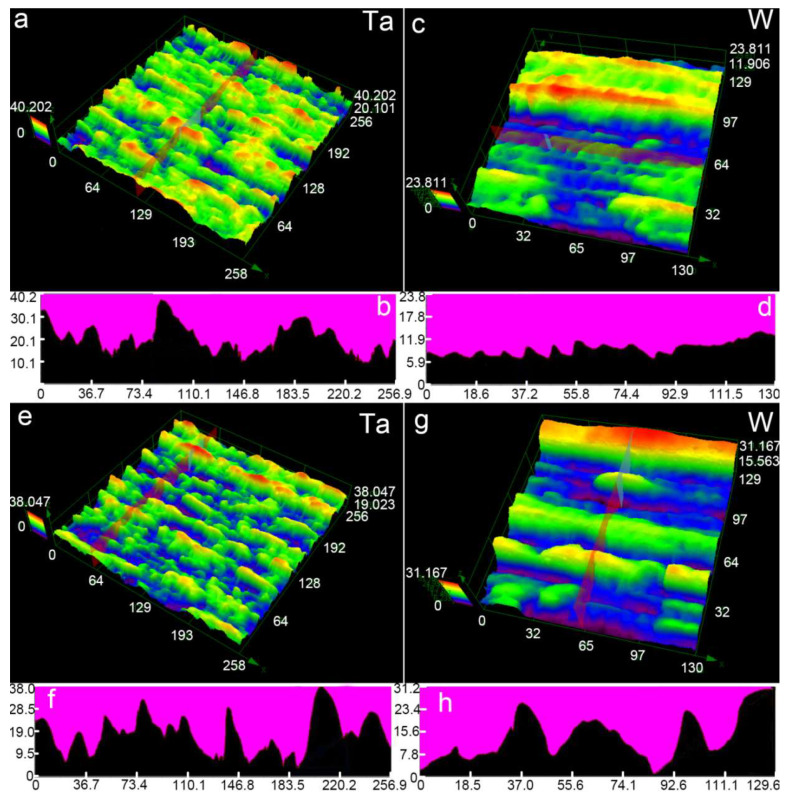
(**a**,**c**,**e**,**g**) Typical 3D morphologies and (**b**,**d**,**f**,**h**) corresponding cross-sectional profiles of hierarchical micro/nanostructures obtained by fs-LAL of (**a**,**b**,**e**,**f**) Ta and (**c**,**d**,**g**,**h**) W in water. (**a**,**e**) and (**c**,**g**) are observed at two different regions from ablated Ta and W samples, and are used to demonstrate the inhomogeneity of the as-prepared surface structures.

Figure 3 displays the 3D morphologies and corresponding cross sectional profiles of the hierarchical micro/nanostructures obtained by fs-LAL of Ta and W in water. The typical structures obtained on Ta surfaces are microstructures with random grooves (Figure 3a,e). The maximal depths of the grooves are ~30 μm, as indicated by the cross-sectional profiles shown in Figure 3b,f. The heights of the microstructures fluctuate heavily, indicating the randomness of the microstructures. In contrast, a crater-rich region is observed on the W surface (Figure 3c). The depths of the microcraters are very shallow, less than 5 μm. Some inhomogeneous grooves with a maximal depth of 23.8 μm are located near the crater-rich region (Figure 3c). The observation of another ablated region, shown in Figure 3g, indicates that the ablation depth on the W surface can still reach 30 μm (the highest region indicated by red color in Figure 3g corresponds to the unablated region). On the W surface, fs-LAL also induced the formation of different groove widths (Figure 3g,h). 

### 3.3. EDS Analysis 

**Figure 4:**
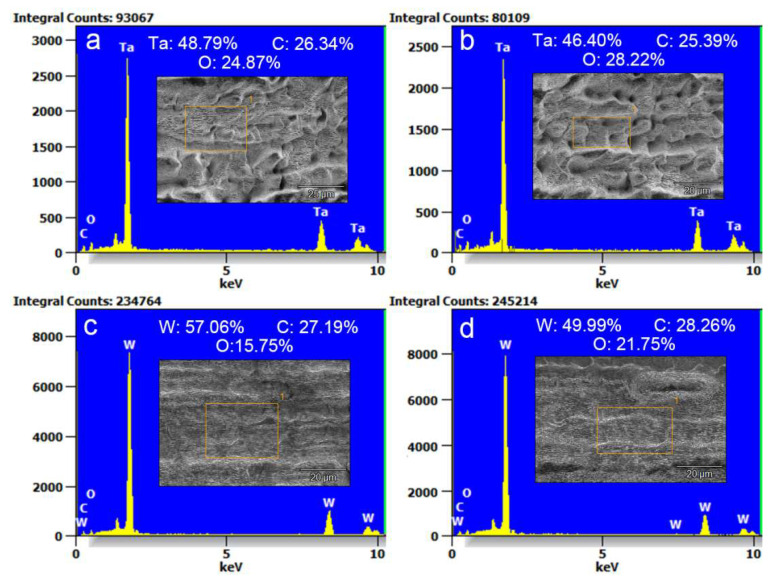
EDS analysis of the structures obtained by fs-LAL of (**a**,**b**) Ta and (**c**,**d**) W in water. The information about the atomic percentages of metal (W or Ta), carbon (C) and oxygen (O) measured from each sample were included in each figure. (**a**,**b**) and (**c**,**d**) are measured at two different regions of surface structures obtained by fs-LAL of Ta and W, respectively.

Figure 4 displays the EDS spectra of structures from the orange rectangles in each inset SEM image obtained by fs-LAL of Ta and W in water. The atomic percentages of metal, carbon and oxygen were tested for two different regions of the hierarchical micro/nanostructures of both Ta and W samples, as shown in Table 2, while also being tested for unablated metal substrates. The atomic percentages of Ta, C and O are 48.79%, 26.34% and 24.87% for the first region of the Ta-structure (Figure 4a, inset image) and 46.40%, 25.39% and 28.22% for the second region (Figure 4b), respectively; and the atomic percentages of W, C and O are 57.06%, 27.19% and 15.75% for the first region of the W-structure and 49.99%, 28.26% and 21.75% for the second region, respectively. The atomic percentages of the non-ablated surfaces of Ta and W are 40.94% Ta, 41.82% C and 17.15% O, and 67.72% W, 20.60% C and 11.69% O, respectively. 

Despite a large difference in carbon percentages between the unablated Ta and W targets, the carbon percentages are almost the same for different structures on all ablated Ta and W samples. However, the oxidation rates are distinctly different for the Ta and W hierarchical structures and are speculated to be associated with different thermal properties of Ta and W, as listed in Table 3. Since it is well known that the formation of UHSFLs is related to the material melting phenomenon during fs-LA [18,31], the melting periods of the ablated materials must strongly depend on the thermal properties of the ablated materials. Both W and Ta have almost the same specific heat of 0.13 and 0.14 J/g K [65]. However, other thermal properties are very different. The thermal diffusivity of ~70 mm^2^/s for W [66] is much higher than that of 24.2 mm^2^/s for Ta [67] at room temperature, and both decrease with an increasing temperature [66]. The thermal conductivities of W and Ta are thus calculated to be 170 and 57 W/m K, respectively. As can be seen, both the thermal diffusivity and thermal conductivity of W are much higher than those of Ta, indicating that W should tend to cool more rapidly than Ta after laser heating. Additionally, the melting temperature of 3422 °C for W is relatively higher than that of 3017 °C for Ta [65,68]. For Ta, the smaller thermal conductivity and thermal diffusivity with a lower melting temperature must endow a relatively longer melting period than W so as to enable the interaction with surrounding oxygen species during fs-LAL. The states of laser pulses such as repetition rates, pulse energy and incident angle may all be modulated by random bubble reflection/refraction [17], so that the thermal states in different positions change a lot. Meanwhile, the shorter periods of the thermal effect of W during fs-LAL may be attributed to a great discrepancy between the oxidation rates at different regions (Figure 4c,d). Nevertheless, despite different oxidation rates in different positions, when compared with the oxygen percentages of the unablated W samples, fs-LAL in water is indeed capable of enhancing the oxidation of the ablated surfaces.

### 3.4. Polarization-Dependent Reflectance 

**Figure 5:**
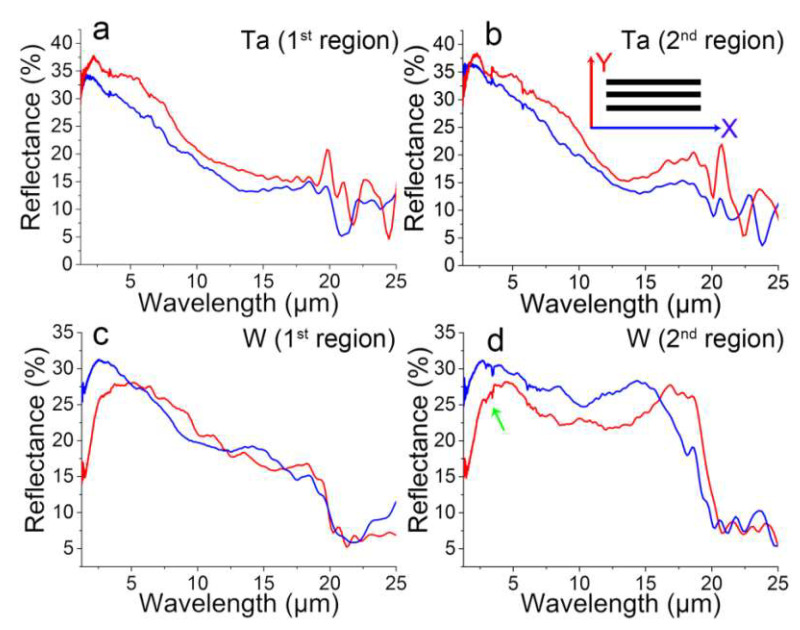
Polarization-dependent reflectance of hierarchical micro/nanostructures at two different positions obtained by fs-LAL of (**a**,**c**) Ta and (**b**,**d**) W in water.

The optical properties of the structures strongly depend on the morphology of the structured surfaces. Anisotropic structures such as parallel grooves [69] and L-shape structures [70] generally possess polarization-dependent reflectance. In this regard, two different regions of the multiscale hierarchical micro/nanostructures of the ablated Ta and W samples were measured for the polarization-dependent reflectance study. Figure 5a,b and Figure 5c,d show the reflective spectra in two different regions on the ablated Ta and W targets in the wavelength range of 1.25–25 μm, respectively. The red curves were measured with the probe light whose polarization direction was perpendicular to the laser scanning direction for fs-LA and was defined as Y-reflectance (as indicated in Figure 5b), while the blue curves were parallel to the horizontal direction (X-reflectance).

Compared to the reflectance, reported elsewhere, of flat hierarchical LSFL/UHSFLs structures obtained by fs-LA in acetone [18], the reflectances in both X- and Y-directions decrease by 60~70% for both the Ta and W samples. This indicates that the endowment of LSFL/UHSFLs hybrid structures on microstructures can significantly enhance the light absorbance and light trapping in the NIR-MIR range, especially in the MIR range. Each X- and Y-reflectance of the Ta measure at two different positions in the wavelength range of 1.25–20 μm shows almost the same characteristics, although they have different reflectance intensities, indicating that the as-prepared Ta structures are similar but with different structure heights (Figure 5a,b). Many small X- and Y-reflection peaks appear in the range of 15–20 μm for Ta, which are ascribed to the existence of LSFLs/HSFLs/UHSFLs [18]. The spectra fluctuations in the wavelength of 20–25 μm of both two Ta samples may be due to inhomogeneous microspikes, as shown in Figure 4a,b.

Figure 5c,d displays variations of the polarization-dependent reflectance on the relatively flat hierarchical structure and the grooved hierarchical micro/nanostructure of W in the wavelength range of 1.25–25 μm, respectively. The reflectance of both samples is almost the same in the range of 1.25–5 μm (Figure 5c,d), with the exception of some small peaks appearing on the grooved sample. The small peaks at ~3.33 μm (as pointed out by a green arrow in Figure 5d) are attributed to the CH stretching from the alkyl groups [71,72]. Such CH stretching mode peaks do not appear on the relatively flat structure (Figure 5c), indicating the structure-dependent surface chemistry induced by fs-LAL. As the wavelength increases from 5 to 20 μm, the X- and Y-reflectances of the relatively flat hierarchical structures look almost same, and both gradually decrease. Broad peaks appear at 14 μm in the X-reflectance and at 18 μm in the Y-reflectance, but the maximal value of each of these two peaks is only slightly higher than the reflectance of the other direction, which is ascribed to the flatness of the micro/nanostructures. Unlike this relatively flat structure, due to the existence of aligned grooves (Figure 5d), the reflectance of the grooved hierarchical micro/nanostructure shows basin-like curves, with new peaks appearing at 15 μm for the X-reflectance and 17.5 μm for the Y-reflectance. Due to the structure difference in the two samples, the polarization-dependent reflectance in the wavelength range of 17.5–25 μm is also different (Figure 5c,d). According to the theory (λ/4 = period of trenches) that the reflectance minima appear at λ = 20 μm in FTIR spectra (Figure 5c,d) [12,13], the trench of the W structures is estimated to be 5 μm in width. From these results, it can be concluded that anisotropic hierarchical micro/nanostructures prepared by fs-LAL can be used to investigate structure-dependent reflectance, a topic that, to date, has been seldom studied in relation to laser-structured surfaces. From the reflectance of the two regions of both the Ta and W structures, it can be concluded that fs-LAL generates more homogenous structures on Ta than on W, which in turn can help explain the huge difference in the region-dependent W structural morphologies shown in Figure 3c,g and oxidation rates shown in Figure 4c,d. 

The EDS analysis (Figure 4) indicates that the ablated surfaces may not be fully oxidized. Moreover, the formation of LIPSSs, including all LSFLs/HSFLs/UHSFLs, is confirmed as resulting from the melting behavior of metal materials [31]. The hierarchical structures should be a mixture of both metal and metal oxides. Since metal oxide materials, including both tantalum oxide and tungsten oxide, are normally insulators [73,74], one should consider composing the as-prepared hierarchical structures out of a lot of metal-insulator-metal (MIM) structures [75], which are good NIR-MIR absorbers due to the fact that their electronic and magnetic resonances occur synchronously at a certain wavelength. These MIM absorbers could contribute to reflectance variations at different wavelengths for different structures on both Ta and W surfaces.

## 4. Conclusions

This work presented the feasibility of developing a hierarchical micro/nanostructure by fs-LAL of Ta and W in water. Due to random light reflection and refraction induced by bubbles, random microstructures, including microgrooves, microcraters and mountain-like microstructures, were formed. All microstructures were fully decorated with hybrid LSFL/HSFL/UHSFLs nanostructures. The periods of LSFLs/HSFLs were in the range of 500–800 nm, and UHSFLs had periods of 30–50 nm. As compared to UHSFL structures obtained on Ta, which had a very limited structure disorder, those prepared on the W surface were very chaotic, especially in the shallow troughs of LSFLs/HSFLs and their outermost ridges, which may be associated with the difference in thermal properties of Ta and W, including the melting temperature, thermal diffusivity and thermal conductivity. Using two different micro/nanostructures on the Ta and W surface as representatives, it was proven that the introduction of a microgroove rendered the polarization-dependent reflectance at wavelengths of 15 and 17.5 μm along the X- and Y-directions, respectively. Ta and W multiscale micro/nanostructures presented in this work possessed lower polarization-dependent reflectances than those of LSFLs/UHSFLs hybrid nanostructures prepared by fs-LAL in acetone [18], which one may ascribe to the presence of microstructures. Integrating hybrid LSFLs/HSFLs/UHSFLs with microstructures into multiscale hierarchical micro/nanostructures can significantly enhance the high absorbance and light trapping in the NIR-MIR range, especially in the MIR range. This work may inspire the preparation of polarization-dependent reflective metasurfaces by fs-LAL, along with studies on the corresponding structure-dependent reflectance.

## Figures and Tables

**Table 1:** Summary of Ta and W LIPSSs obtained by laser ablation in air and liquids.

Sample	Laser Parameters	Environment	LIPSSs	Ref
Ta, W	457 fs, 1045 nm, 100 kHz, 600 mW, 6 μJ/pulse, 66.12 J/cm^2^, 1 mm/s	water	400–600 nm 30–50 nm	This work
Ta, W	457 fs, 1045 nm, 100 kHz, 600 mW, 6 μJ/pulse, 66.12 J/cm^2^, 1 mm/s	acetone	370–600 nm 20–60 nm	[18]
Ta	500 fs, 1030 nm, 100 kHz, 2–6 μJ/pulse, 0.30 J/cm^2^, 200 mm/s	air	780 ± 48 nm	[52]
W	30 fs, 800 nm, 1 kHz, 0.6–2.5 J/cm^2^	ethanol	310–340 nm	[55]
W	70 fs, 800 nm, 1 kHz, pulse delay 0–14 ps, 1 J/cm^2^	ethanol	310–370 nm	[56]
W	180 fs, 800 nm, other parameters cannot be found	ethanol	350 nm	[54]
W	200 fs, 2 kHz, 775 and 387 nm, 0.11 and 0.1 J/cm^2^	air	400–460 nm 130–230 nm	[57]
W	160 fs, 800 nm, 10 Hz, 1.1–34 μJ/pulse, 0.2–1 J/cm^2^	air	600–700 nm	[58]
W	50 fs, 800 nm, 0.09 J/cm^2^	air	800 nm	[59]
W	30 fs, 800 nm, 1 kHz, 0.09–1.81 J/cm^2^	air	634 ± 48 nm	[60]
W	33 fs, 800 nm, 1 kHz, 0.4–3.2 μJ/pulse	air	350–600 nm	[26]
W	33 fs, 800 nm, 1 kHz, 3–12 J/cm^2^	air	~550 nm	[49]
W	90 fs, 800 nm, 80 MHz, 18–24 mW	air	150–185 nm	[53]
W	65 fs, 400/800 nm, 1 kHz, 0.35/0.37 J/cm^2^	air	289/542 nm	[61]
W	150 fs, 30 μJ/pulse, 0.77 J/cm^2^	air	~600 nm	[62]
W	140 fs, 400 nm, 1 kHz, 0.5 J/cm^2^	air	304–309 nm	[63]
W	50 fs to 8 ps, 800 nm, 1 kHz, 0.1–1.2 mm/s, 0.8–6.2 J/cm^2^, 0.1–1.2 mm/s	air	450–690 nm	[64]

**Table 2:** Atomic percentages of non-ablated Ta/W and two regions of ablated Ta and W.

Sample	Metal (Ta/W)	Carbon	Oxygen
Non-ablated Ta	40.94	41.82	17.15
Ta ablated region 1	48.79	24.84	26.34
Ta ablated region 2	46.40	25.39	28.22
Non-ablated W	67.72	20.60	11.69
W ablated region 1	57.06	27.19	15.75
W ablated region 2	49.99	28.26	21.75

**Table 3:** Thermal properties of metal Ta and W.

Material	Melting Temperature (°C)	Thermal Diffusivity (MM^2^/S)	Thermal Conductivity (W/m·K)	Specific Heat (J/g K)
Ta	3017	24.2	57	0.14
W	3422	~70	170	0.13
